# COVID-19 Death Reporting Inconsistencies and Working Lessons for Low- and Middle-Income Countries: Opinion

**DOI:** 10.3389/fmed.2021.595787

**Published:** 2021-02-17

**Authors:** Garumma Tolu Feyissa, Lemi Belay Tolu, Alex Ezeh

**Affiliations:** ^1^Dornsife School of Public Health, Drexel University, Philadelphia, PA, United States; ^2^Department of Obstetrics and Gynecology, Saint Paul's Hospital Millennium Medical College, Addis Ababa, Ethiopia

**Keywords:** mortality, corona-virus 2, SARS CoV-2, COVID-19, severe acute respiratory coronavirus 2, excess mortality

## Introduction

The corona-virus disease 2019 (COVID-19) has caused terrible health, social and economic impacts in the entire world. Thousands have died of the virus since its identification in Wuhan province of China. Deaths from the corona-virus disease 2019 (COVID-19) pandemic might arise both in those infected (direct effects), as well as those affected (not infected, but affect indirectly) by altered access to health services; the physical, psychological, and social effects of social distancing; and economic changes. Yet there is no consensus on what to consider as COVID-19 death. In some countries, the reported number of COVID-19 deaths do not include deaths attributed to underlying conditions even when the cases had tested positive for COVID-19 (1). In other countries, such cases are included as COVID-19 deaths. Other countries go even further to include suspected cases in their reports of COVID-19 mortality (2). These factors confuse the comparison of case fatality rates across countries, mainly in low- and middle-income countries (LMICs), where data recording system and vital registrations are weak. These differences in the reporting of COVID-19 deaths also make it difficult to generate robust evidence to inform policies and strategies to reduce excess mortality from the pandemic. Because of the lack of structured civil registry systems, some low-income countries might be claiming low mortalities related to COVID-19, because of unreported deaths. This may result in unnecessary over confidence or claim of control of the pandemic, which may further lead to low preventative measures among the community and policy makers.

## Reporting Direct COVID-19 Deaths

COVID-19 deaths are mainly defined in two ways. The first method uses clinically confirmed or probable COVID-19 cases. The World Health Organization (WHO) definition concurs with this method. countries such as Belgium, Canada, France, and Germany use this method of reporting. The WHO defines death from COVID-19 as “any death resulting from a clinically compatible illness resulting from a probable or confirmed diagnosis of COVID-19, unless there is a clear alternative cause of death that cannot be related to COVID-19 disease (e.g., trauma)” (3). For example, patients with COVID-19 who die from other diseases or accidents are not certified as COVID-19 death (3). As per the WHO definition, to be considered as COVID-19 death, there should not be a period of complete recovery (from COVID-19) between illness and death. This is also referred to as direct death from COVID-19. Such deaths are not caused by other diseases. According to the WHO reporting protocol, COVID-19 deaths are counted independently of pre-existing conditions that might have been exacerbated by morbidity from the severe course of COVID-19. The WHO protocol also recommends that suspected cases of COVID-19 should be counted as COVID-19 death if there is no other alternative explanation for the death (3). The second method relies exclusively on a laboratory test results. Countries, such as Austria, Italy, Spain, the Netherlands, and the United Kingdom use this method of reporting (4).

All things being equal, countries using the WHO definition will generally capture more COVID-19-associated deaths compared to those using exclusively positive laboratory tests for COVID-19. There are many reasons to expect that reports of COVID-19-associated deaths based on positive test results will generally underreport COVID-19 deaths. First, there are wide variations in COVID-19 testing policies across countries. For instance, some countries restrict testing to population groups with severe symptoms. Second, testing capacities vary across countries and across localities within countries. The third challenge is related to the low test sensitivity of polymerase chain reaction (PCR), which may result in false negative cases (5). This method could limit the reporting of COVID-19 deaths to hospital deaths. This will likely result in a relatively higher case-fatality rates because of the small sample of the population having access to tests. The European Center for Disease Prevention and Control (ECDC) endorses the WHO definition and also recommends that the European countries monitor total, as well as excess mortality related to the pandemic (6).

## Reporting Excess Deaths Associated With COVID-19

Excess mortalities are defined as “the difference between the observed numbers of deaths in specific time periods and expected numbers of deaths in the same time periods” (7). This method compares weekly deaths with previous trends in deaths in specific locality (8). Excess deaths associated with COVID-19 comprise not only deaths directly caused by COVID-19 but also indirect deaths (i.e., deaths of persons not infected with COVID-19). Indirect causes of death include deaths caused by distorted access to health care services; the negative impact of pandemic on physical, psychological, and social well-being and negative impact of social distancing; and economic challenges. Such types of deaths are expected to increase significantly in LMICs because of the already fragile healthcare system and limited expansion of alternative healthcare services such as online or phone consultation and prescription services during the lockdown. This category also encompasses the deaths that are missed by some case definitions. It includes deaths resulting from pre-existing health conditions, such as hypertension, diabetes, cardiovascular disorders, malnutrition, and other conditions related to poverty. Such report of mortality is indispensable for systematic planning even during the post-COVID-19 period. In the past, indirect effects of epidemics and pandemics have resulted in a remarkable incidence of deaths at different times in different parts of the world. Evidence from the outbreak of Ebola virus during the year 2013 to 2016 can be mentioned as a good example of the indirect effects that such crises can have. For instance, an analysis of the Sierra Leone's Health Management Information System (HMIS) reported an estimated 3,600 maternal deaths, neonatal deaths, and stillbirths attributed to disruption of the health system related to the outbreak. This figure is approximately equal to the total direct deaths of Ebola for Sierra Leone (9).

Similarly, early estimates have indicated that the disrupted healthcare services and limited access to food resulting from the COVID-19 lock-down would result in 1,157,000 excess child mortalities and 56,700 excess maternal mortalities over a 6 months period in LMICs (10). Another estimate has shown that a 10% reduction of healthcare services in LMICs would result in excess maternal mortalities of 1,000 and 28,000 just only from unsafe abortion and obstetric complications, respectively. The same report estimated 168,000 additional newborn deaths (11).

Taking the variations in reporting COVID-19 deaths and limitations in the low sensitivity of existing PCR tests into account, it is imperative that excess deaths associated with COVID-19 be reported especially in low-and-middle income countries. The advantage of such a report is that it addresses inconsistencies resulting from the disparities in the reporting of COVID-19 mortalities across countries and locations. Excess mortalities associated with COVID-19 also counts deaths of suspected cases of COVID-19. Hence, it provides a complete depiction of the impact of the crisis created by the pandemic. Furthermore, it lays adequate ground for tracking of the impact of different types of policies and interventions and natural progress of the pandemic over time. In developed countries, testing capacity has increased since the beginning of the pandemic. This will potentially improve the accuracy of mortality reports by reducing unconfirmed and/or suspected deaths. On the other hand, developing countries are still facing structural barriers, such as socio-economic and political problems and limited institutional capacity limiting their testing capacities. If feasible, representative surveys specifically designed for reporting of COVID-19 may be conducted to minimize these problems.

## Conclusion and Recommendations

The COVID-19 mortalities are mainly reported in either of two ways: either diagnosis-based or test-based. The WHO reporting format, which is diagnosis based, is expected to address complete picture of COVID-19-associated deaths. However, the variations that exists in the level of implementation of WHO guidance may be challenging to this method of reporting COVIID-19 mortality. Moreover, death certificate reporting may take long time, further compromise the timeliness of the reports. Poor vital registration system and weak data management in low-and-middle income countries further complicate the challenges related to the reporting COVID-19 associated mortalities. With these considerations, we recommend that LMICs should use the WHO international guidelines for certification and classification of COVID-19 deaths. Given that most LMICs have only limited testing capacity and data tracking capacity, estimating excess death can provide complete picture of the impact of the pandemics of COVID-19. Therefore, LMICs should report excess mortality, which includes both direct and indirect causes of deaths during the pandemic of COVID-19. This is critical to appropriately evaluate the impact of different policies and strategies and to develop context-based strategies to mitigate the negative impacts of the COVID-19 pandemic. Where feasible, well-designed representative surveys may help to minimize the challenges associated with the reporting of COVID-19 associated deaths.

## Author Contributions

All authors contributed equally to the content and read and approved the final version of the manuscript.

## Conflict of Interest

The authors declare that the research was conducted in the absence of any commercial or financial relationships that could be construed as a potential conflict of interest.
